# Osteoblast-specific overexpression of complement receptor C5aR1 impairs fracture healing

**DOI:** 10.1371/journal.pone.0179512

**Published:** 2017-06-14

**Authors:** Stephanie Bergdolt, Anna Kovtun, Yvonne Hägele, Astrid Liedert, Thorsten Schinke, Michael Amling, Markus Huber-Lang, Anita Ignatius

**Affiliations:** 1 Institute of Orthopedic Research and Biomechanics, University of Ulm, Ulm, Germany; 2 Department of Osteology and Biomechanics, University Medical Center Hamburg-Eppendorf, Hamburg, Germany; 3 Institute of Clinical and Experimental Traumaimmunology, University of Ulm, Ulm, Germany; University of Zaragoza, SPAIN

## Abstract

The anaphylatoxin receptor C5aR1 plays an important role not only in innate immune responses, but also in bone metabolism and fracture healing, being highly expressed on immune and bone cells, including osteoblasts and osteoclasts. C5aR1 induces osteoblast migration, cytokine generation and osteoclastogenesis, however, the exact role of C5aR1-mediated signaling in osteoblasts is not entirely known. Therefore, we hypothesized that osteoblasts are essential target cells for C5a and that fracture healing should be disturbed in mice with an osteoblast-specific C5aR1 overexpression (Col1a1-C5aR1). Osteoblast activity *in vitro*, bone phenotype and fracture healing after isolated osteotomy and after combined osteotomy with additional thoracic trauma were analyzed. The systemic and local inflammatory reactions were analyzed by determining C5a and IL-6 concentrations in blood, bronchoalveolar lavage fluid and fracture callus and the recruitment of immune cells. *In vitro*, osteoblast proliferation and differentiation were similar to wildtype cells, and phosphorylation of p38 and expression of IL-6 and RANKL were increased in osteoblasts derived from Col1a1-C5aR1 mice. Bone phenotype and the inflammatory reaction were unaffected in Col1a1-C5aR1 mice. Fracture healing was significantly impaired as demonstrated by significantly reduced bone content, bone mineral density and flexural rigidity, possibly due to significantly increased osteoclast numbers. C5aR1 signaling in osteoblasts might possibly affect RANKL/OPG balance, leading to increased bone resorption. Additional trauma significantly impaired fracture healing, particularly in Col1a1-C5aR1 mice. In conclusion, the data indicate that C5aR1 signaling in osteoblasts plays a detrimental role in bone regeneration after fracture.

## Introduction

In recent years, evidence has accumulated that the complement system, an essential part of innate immunity, is involved in the regulation of bone turnover and in the pathogenesis of bone disorders, including fracture healing. However, the underlying mechanisms are currently poorly understood [1–3].

The complement system, a network of more than 30 soluble and membrane-bound molecules, serves as a powerful danger-sensing system, which is activated by exogenous and endogenous danger molecules [4]. It can be activated intrinsically by the alternative, lectin or classical pathway or extrinsically by serine proteases, including thrombin of the coagulation system [4,5]. All pathways lead to the generation of the anaphylatoxin C5a, which crucially triggers the inflammatory response of immune cells. For example, C5a induces the migration of inflammatory cells, the degranulation of mast cells and the release of inflammatory cytokines and regulates apoptosis [6–8]. The pro-inflammatory actions of C5a are mediated by its receptor C5aR1, a classical G protein-coupled receptor, expressed on the plasma membrane of a variety of cells, particularly immune cells, including neutrophils, eosinophils and basophils [6,9]. However, C5aR1 is also expressed on non-immune cells, including, among others, endothelial cells and neurons, suggesting that complement-mediated signaling is not limited to inflammatory cells [8]. We and others have demonstrated that C5aR1 is also strongly upregulated during osteogenic differentiation of mesenchymal stem cells (MSCs) and induced osteoblast migration and the production of pro-inflammatory cytokines, including interleukin (IL)-6 and IL-8 *in vitro* [1,10,11]. Notably, C5a also enhanced the expression of receptor activator of nuclear factor-κB ligand (RANKL) in osteoblasts, a key stimulator of osteoclastogenesis, indicating that C5a may indirectly induce osteoclastic bone resorption [12]. C5aR1 is also expressed on osteoclasts and can directly regulate their differentiation and activity [1,13]. Taken together, these data indicate that the activation of the C5a/C5aR1 axis could modulate bone cell activity and their immune-modulatory response [3]. The C5aR1 axis in bone cells might be particularly important under inflammatory conditions after bone injury. Accordingly, we demonstrated that C5aR1 was strongly upregulated in the periosteum, where precursor cells already start to proliferate and undergo osteogenic differentiation during the early phase after bone fracture [1]. These osteoblasts may modulate the local immune response after stimulation with C5a, which is locally and, depending on trauma severity, systemically generated as a response to damage-associated molecular patterns. Furthermore, C5aR1 was strongly expressed by bone-forming osteoblasts in the fracture callus over the entire healing period [1], indicating that osteoblasts may serve as target cells for C5a not only in the early inflammatory phase but also later during bone repair. Confirming this, C5-deficient mice displayed disturbed bone formation in the fracture callus [2]. However, the specific role of the C5a-C5aR1-interaction in osteoblasts during bone healing is currently poorly understood.

Therefore, we investigated the function of the C5a/C5aR1 axis in osteoblasts during bone repair using transgenic mice with an osteoblast-specific C5aR1 overexpression. We evaluated fracture healing in a model of uncomplicated isolated fracture and in a model of impaired healing by combining the fracture with an additional thoracic trauma, which is associated with local and systemic complement activation and severe inflammation [14,15]. We hypothesize that if osteoblasts were important target cells for C5a, fracture healing would be disturbed.

## Materials and methods

### Mouse model

The mouse experiments were performed according to the international regulations for the care and use of laboratory animals and approved by the Local Ethical Committee (Regierungspräsidium Tübingen, GER, Reg.Nr. 1096). All mice were housed in groups of up to four mice per cage with a 14 h light and 10 h dark cycle and received a standard mouse feed (ssniff^®^ R/M-H, V1535-300, Ssniff, Soest, Germany) and water *ad libitum*. Mice with an osteoblast-specific overexpression of C5aR1 (Col1a1-C5aR1) were generated as follows: The open reading frame encoding C5aR1 was placed under the control of the osteoblast-specific 2.3 kb *Col1a1* promotor fragment. To confirm C5aR1 overexpression, Col1a1-C5aR1 mice were genotyped using the forward primer 5-ACTACATCCTGGTCATCATCCTGC-3 and the reverse primer 5-CCAGCAGGAAACGGTCGGCA-3. Wildtype littermates (WT) were used as controls.

### Cultivation of primary mouse osteoblasts

Primary osteoblasts were isolated from long bones of 8- to 12-week-old WT and Col1a1-C5aR1 mice. Briefly, long bones were harvested and bone marrow was flushed out using phosphate-buffered saline (PBS, Biochrom, Berlin, Germany). The diaphyses were cut into small pieces, which were digested with collagenase (1 mg/ml collagenase type II (125 U/mg, Sigma-Aldrich, Taufkirchen, Germany)) for 2 h at 37°C and then washed twice with PBS. For osteoblast expansion, bone fragments were cultivated in culture medium (α-MEM, Biochrom) supplemented with 10% heat-inactivated fetal calf serum (Gibco, Darmstadt, Germany), 100 U/ml penicillin/streptomycin (Gibco), 1% L-glutamine (PAN-Biotech, Aidenbach, Germany) and 0.5% Fungizone^TM^ (Amphotericin B, Gibco) at 37°C and 5% CO_2_ atmosphere.

To induce osteogenic differentiation, osteoblasts were seeded in 24-well plates (CELLSTAR®, Greiner Bio-One) at a density of 0.5 × 10^4^ cells/cm^2^ in the presence of 0.2 mM ascorbate-2-phosphate (Sigma-Aldrich) and 10 mM β-glycerophosphate (Sigma-Aldrich) for 14 d. Alkaline-phosphatase staining (Sigma-Aldrich) was performed to confirm osteogenic differentiation. To investigate whether the differentiation capacity was influenced by C5a, 0.1 μg/ml of recombinant mouse C5a (R&D Systems, Minneapolis, USA) was added to the differentiation medium. To investigate whether C5a can induce a local inflammatory response in osteoblasts after osteogenic differentiation, cells were stimulated with 0.1 μg/ml C5a for 6 or 24 h and analyzed by western blot and real-time quantitative PCR (RT-qPCR), respectively.

Osteoblast proliferation was assessed using the BrdU Cell Proliferation Kit (Cell Signaling Technology, Danvers, MA, USA) according to manufacturer’s recommendations. Briefly, osteoblasts were seeded in 96-well plates at a density of 1.6 × 10^4^ cells/cm^2^. BrdU detection antibody (1:100) was added to culture medium for 24 h at 37°C. Cells were then incubated with a horseradish peroxidase (HRP)-conjugated secondary antibody for 1 h at RT, before adding 3,3,5,5-Tetramethylbenzidin substrate for 25 min at RT. The cells were analyzed by spectrophotometry at 450 nm.

### Reverse transcription and real-time quantitative PCR

Total RNA was isolated from cells using the RNeasy Mini Kit (Qiagen, Hilden, Germany) according to the manufacturer’s instructions and cDNA was transcribed from 1 μg RNA using the Omniscript RT Kit (Qiagen). The StepOnePlus^TM^ Real-Time PCR System (Applied Biosystems, Darmstadt, Germany) and the Platinum^®^ SYBR^®^ Green qPCR SuperMix-UDG (Invitrogen ThermoFisher Scientific, Waltham, MA, USA) were used for cDNA amplification and quantification according to the manufacturer’s instructions. Samples were initially incubated at 50°C and at 95°C, for 2 min each, followed by 40 cycles with the following cycling conditions: 95°C for 15 s and 60°C for 1 min. The amplification protocol was terminated with a melting-curve step at 95°C for 15 s, followed by 60°C for 1 min. Gene expression was analyzed relative to the housekeeping gene glyceraldehyde-3-phosphate dehydrogenase (GAPDH) using the ΔΔC_t_ method with PCR-efficiency correction using LinRegPCR software as described previously [16]. All primers were purchased from Invitrogen or Thermo Electron (Ulm, Germany) (Table 1).

**Table 1:** Primer sequences.

Target Gene	Forward Primer Sequence (5'–3')	Reverse Primer Sequence (5'–3')
**AP *(Alpl)***	GCT GAT CAT TCC CAC GTT TT	GAG CCA GAC CAA AGA TGG AG
**OCN *(Bglap)***	GCG CTC TGT CTC TCT GAC CT	ACC TTA TTG CCC TCC TGC TT
**BSP *(Ibsp)***	GAA GCA GGT GCA GAA GGA AC	GAA ACC CGT TCA GAA GGA CA
**GAPDH *(Gapdh)***	ACC CAG AAG ACT GTG GAT GG	GGA TGC AGG GAT GAT GTT CT
**IL-6 (*Il6*)**	TCC TTC CTA CCC CAA TTT CC	GCC ACT CCT TCT GTG ACT CC
**RANKL *(Tnfsf11)***	ATC ATG AAA CAT CGG GAA GC	CTT GGG ATT TTG ATG CTG GT
**OPG *(Tnfrsf11b)***	CTG CCT GGG AAG AAG ATC AG	GCT CGA TTT GCA GGT CTT TC

Protein name with gene name in bracket. AP: alkaline phosphatase, OCN: bone gamma carboxyglutamate protein/osteocalcin, BSP: bone sialoprotein, GAPDH: glyceraldehyde-3-phosphate dehydrogenase, IL-6: interleukin 6, RANKL: receptor activator of nuclear factor-κB ligand, OPG: osteoprotegerin.

### Western blot

Osteoblasts were lysed in Pierce® RIPA buffer (ThermoFisher Scientific, Waltham, MA, USA), containing protease and phosphatase inhibitors (ThermoFisher Scientific). We resolved 10 μg of total protein on a 10% sodium dodecyl sulfate-polyacrylamide gel electrophoresis and transferred it to a nitrocellulose membrane (Amersham^TM^ Protran^TM^ 0.2 m NC, GE Healthcare, Chicago, IL, USA). Antibody blocking was performed in Tris-buffered saline with Tween20 (TBS-T) with 3% bovine serum albumin for 1 h at room temperature (RT). Primary rat anti-mouse C5aR (CD88) (MA1-81761, 1:1000, ThermoFisher Scientific), rabbit anti-mouse p-Erk1/2 (4370P, 1:1000, Cell Signaling Technology), rabbit anti-mouse Erk1/2 (4695P, 1:1000, Cell Signaling Technology), rabbit anti-mouse p-p38 (4511P, 1:1000, Cell Signaling Technology), rabbit anti-mouse p38 (8690P, 1:1000, Cell Signaling Technology) and a rabbit anti-mouse GAPDH antibody (2118, 1:2000, Cell Signaling) were incubated overnight at 4°C, followed by incubation with a secondary HRP-coupled anti-rat or anti-rabbit antibody (1:15,000, Cell Signaling Technology), respectively, at RT for 1 h. WesternBright^TM^ Quantum or WesternBright^TM^ ECL chemiluminescent HRP substrate (both Advansta, CA, USA) were added to the membranes for 2 min at RT and the luminescent signal was captured using the Fusion Molecular Imaging System (Vilber Lourmat, Eberhardzell, Germany) or by membrane exposure to X-ray film.

### Animal studies

To investigate whether osteoblast-specific C5aR1 overexpression influences bone development and bone mass, the skeleton of 12- and 55-week-old male WT and Col1a1-C5aR1 mice was analyzed by micro-computed tomography (μCT) and histomorphometry as described below. Mice received subcutaneous injections of alizarin red and calcein green (both 30 mg/kg) (both Sigma, Steinheim, Germany) 3 and 12 days before bone harvesting to determine dynamic bone formation.

To study fracture healing, 12-week-old male Col1a1-C5aR1 mice and WT littermates received a standardized femur osteotomy as described previously [17]. Briefly, mice were anesthetized with 2% isoflurane (Forene, Abbott, Wiesbaden, Germany) and an external fixator (axial stiffness 3 N/mm, RISystem Ind., Davos, Switzerland) was attached to the femur using four Schanz-screws. An osteotomy gap was created between the two inner pins using a wire saw of 0.4 mm diameter (RISystem Ind.). Immediately after the osteotomy, half of the mice received an additional thoracic trauma to induce a bilateral, isolated lung contusion via a blast-wave generator centered 2 cm above the middle of the thorax as described previously [14,18]. For pain medication, all mice received 25 mg/l tramadol hydrochloride (Tramal®, Gruenenthal GmbH, Aachen, Germany) in the drinking water 1 d before and up to 3 d after surgery. Mice were sacrificed 3 h, 1 d, 3 d, 14 d, 21 d and 25 d after surgery using an overdose of CO_2_ and subsequent heart puncture with blood withdrawal. Blood for serum/plasma, bronchoalveolar lavage (BAL) fluid and femur were harvested for further analyses.

### Analysis of inflammatory mediators in blood, BAL fluids and callus homogenates

Blood was harvested 3 h after surgery in microvettes (Sarstedt AG & Co., Nümbrecht, Germany) and centrifuged at 10,000 × *g* for 10 min for plasma collection or at 2800 × *g* for 10 min for serum collection and stored at −80°C. At the same time point, BAL fluid was collected by flushing the lungs with 500 μl of ice-cold PBS. After centrifugation at 4000 × *g* for 10 min, the supernatant was stored at −80°C [19]. Fractured femurs were explanted 3 h after surgery, shock-frozen in liquid nitrogen and stored at −80°C. The fracture hematoma was collected in 150 μl lysis buffer (10 mM Tris pH 7.5, 10 mM NaCl, 0.1 mM ethylenediaminetetraacetic acid, 0.5 mM Triton X-100, 0.02% NaN_3_, 0.2 mM phenylmethylsulfonyl fluoride) with protease inhibitors (1:100 vol/vol Sigma-Aldrich) and homogenized. After incubation for 30 min on ice, the samples were centrifuged for 30 min at 10,000 × *g* at 4°C. The protein concentration in the supernatant was determined using the Pierce^TM^ BCA Protein Assay Kit (Fisher Scientific GmbH, Schwerte, Germany) according to manufacturer’s protocol and lysates were stored at −80°C. C5a concentrations were determined in plasma, callus lysates and BAL fluid and IL-6 was determined in serum using mouse enzyme-linked immunosorbent assay (ELISA) kits (C5a: R&D Systems, 1:100, 1:50 and 1:100; respectively; IL-6: BD Biosciences, Singapore, 1:2). IL-6 concentrations in callus lysates and BAL fluid were determined using a mouse Multiplex-ELISA kit (Bio-Plex Pro Cytokine Assay, Bio-Rad, Hercules, CA, USA). Data were analyzed using the standard curve of cytokine standards and values below the detection limit of the assay were set to zero.

### Micro-computed tomography

Following fixation in 4% formalin, the femurs were scanned by micro-computed tomography (μCT) using a Skyscan 1172, (Kontich, Belgium) at 50 kV, 200 μA and a resolution of 8 μm [14]. Analysis of μCT data was performed according to the guidelines of Bouxsein and colleagues [20] and the standard American Society for Bone and Mineral Research (ASBMR) guidelines for μCT analysis [21]. For bone phenotyping, the volumes of interest (VOIs) were defined as follows: 168 μm height in the mid-diaphysis of the cortical bone and for the trabecular bone in the distal part of the femur 200 μm above the growth plate and 280 μm height. For analysis of fracture healing, the fracture gap was defined as the VOI. Global thresholding (641.9 mg hydroxyapatite/cm^3^) was used to distinguish between mineralized and non-mineralized tissue to calculate tissue mineral density and trabecular thickness, number and separation (CTAnalyser, Skyscan) [22]. Bone mineral density was determined without thresholding.

### Histology and immunohistochemistry

Femurs were fixed in 4% buffered formaldehyde and embedded in paraffin for Safranin-O staining (14 d after surgery) or in polymethylmethacrylate for Giemsa staining (21 d and 25 d after surgery) and bone phenotyping. Longitudinal sections of the fractured femur were cut in the anterior-posterior direction. Bone, cartilage and fibrous tissue areas were assessed using image-analyzing software (MMAF Version 1.4.0 MetaMorph®, Leica, Heerbrugg, Switzerland) and OsteoMeasure® image-analysis software (OsteoMetrics, Inc., GA, USA) was used to determine the number of osteoblasts in toluidine blue-stained sections, the number of osteoclasts in tartrate-resistant acid phosphatase (TRAP)-stained sections and the bone formation rate according to the guidelines of the ASBMR [21].

Sections of the fractured femurs were immunostained 1 and 3 d after surgery for neutrophils (Ly-6G^+^, #127632, BioLegend, San Diego, CA, USA), macrophages (F4/80^+^, Bio-Rad AbD Serotec GmbH, Puchheim, Germany) and T-lymphocytes (CD8^+^, AM11118PU-S, Acris, Herford, Germany), and 14 d after surgery for Runx2 (cs8486, Cell Signaling), osteocalcin (orb77248, Biorbyt Ltd, Cambridge, UK), RANKL (sc-7628, Santa Cruz, Dellas, Texas, USA) and osteoprotegerin (OPG, APO6631PU-N, Acris). After deparaffinizing the 4% buffered formaldehyde-fixed femur sections in xylene, they were rehydrated and blocked (F4/80^+^, Runx2 and osteocalcin with 5% goat serum; Ly6-G^+^, CD8^+^ and OPG with 10% goat serum) for 1 h at RT. Sections were incubated with primary antibodies (Ly6-G^+^ 1:300, F4/80^+^ 1:500, CD8^+^ 1:500, Runx2 1:50, osteocalcin 1:200, RANKL 1:100, OPG 1:400) at 4°C overnight except for CD8^+^ for 2 h at RT and then all for 1 h at RT with respective secondary antibodies (goat anti-rat or goat anti-rabbit, biotinylated, 1:200, Life Technologies, Carlsbad, CA, USA). To detect the antibodies, ABC kit and NovaRED substrate (both Vector laboratories Inc., Burlingame, CA, USA) were applied according to the manufacturer’s protocol, followed by counterstaining with hematoxylin. For analysis under a light microscope (Leica DMI6000 B, Leica, Heerbrugg, Switzerland), the region of interest was defined as the periosteal callus between the inner pins of the fixator. Macrophage staining was analyzed in the marrow cavity in direct proximity to the fracture gap.

### Biomechanical testing

Osteotomized and intact femurs were explanted 21 d and 25 d after surgery and the flexural rigidity was analyzed by a non-destructive, three-point bending test using a material testing machine (Z10, Zwick GmbH, Ulm, Germany) [17]. Briefly, an axial bending load with a force of up to 4 N was applied on top of the callus or on the mid-point of the diaphysis of the intact femur. The flexural rigidity (EI) was calculated from the slope of the force deflection curve. The EI of the fractured femur was related to the contralateral femur of the same animal [17].

### Statistical analysis

Proliferation and osteogenic differentiation of primary osteoblasts was analyzed in 5–6 independent experiments. Bone phenotype and fracture healing were determined in 5–8 mice per genotype and time point.

Groups were tested for normal distribution using the Shapiro-Wilk test and then compared by either Kruskall-Wallis and Dunn’s *post-hoc* test or by one-way analysis of variance and Fishers LSD *post-hoc* test using GraphPad Prism 6 (GraphPad Software Inc., La Jolla, CA, USA). Data are presented as mean ± standard deviation. The level of significance was set at p< 0.05.

## Results

### Osteoblast-specific overexpression of C5aR1 did not cause a bone phenotype

**Fig 1:**
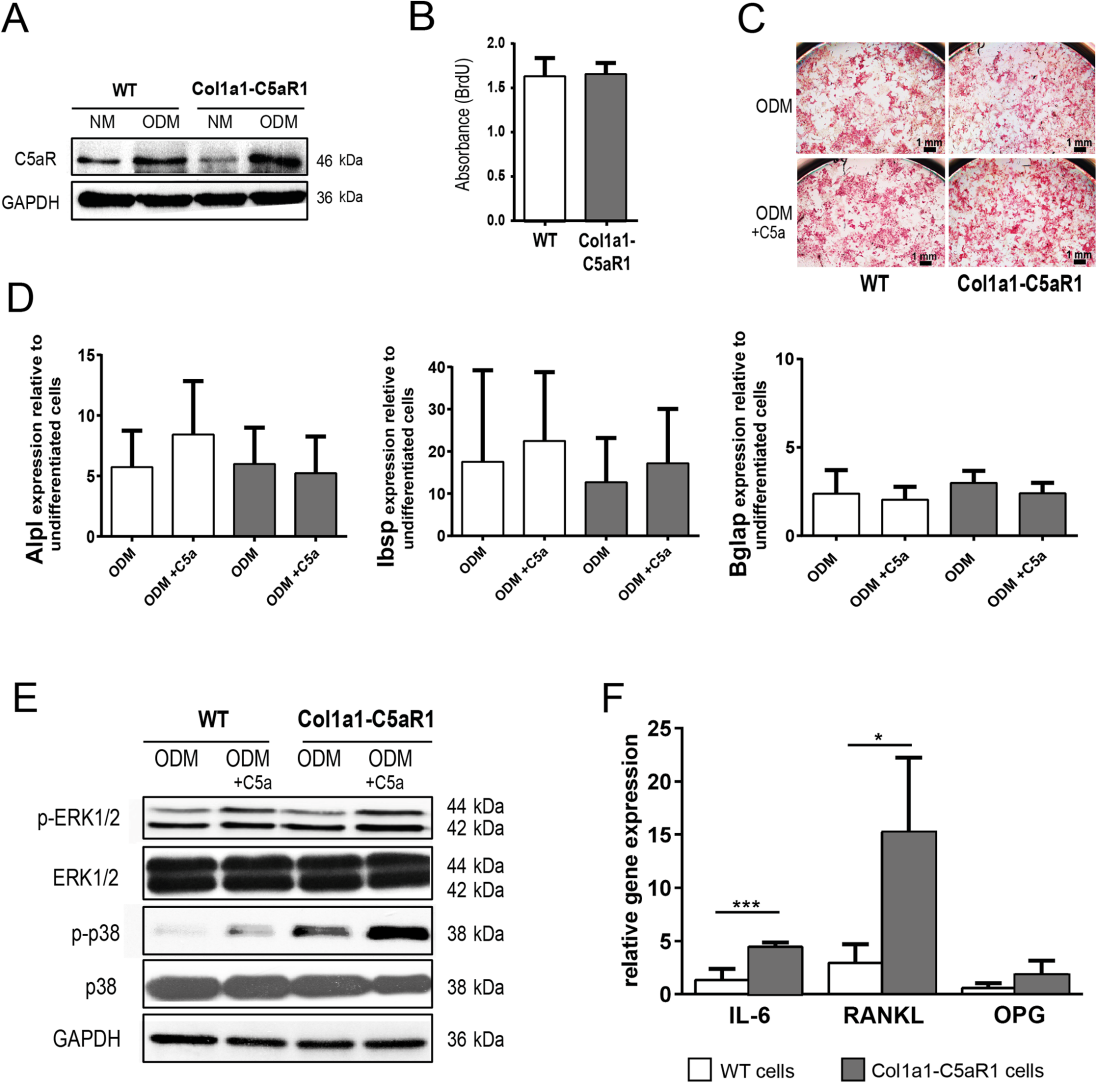
*In vitro* analysis of WT and Col1a1-C5aR1 osteoblasts. (A) Western blot showing C5aR1 expression in primary osteoblasts of wildtype (WT) and Col1a1-C5aR1 mice after cultivating for 14 d in normal proliferation medium (NM) and osteogenic differentiation medium (ODM). (B) Cell proliferation of WT and Col1a1-C5aR1 osteoblasts determined by BrdU incorporation. (C) Osteogenic differentiation capacity of osteoblasts assessed by alkaline-phosphatase staining after cultivating for 14 d in ODM with or without C5a and (D) by the expression of the osteogenic marker genes *Alpl* (alkaline phosphatase), *Ibsp* (bone sialoprotein) and *Bglap* (bone gamma carboxyglutamate protein/osteocalcin). (E) Western blot showing increased expression of phospho (p)-ERK 1/2 and phospho (p)-p38 after osteoblast stimulation with C5a for 6 h. F) Relative gene expression of interleukin (IL)-6, receptor activator of NF-κB ligand (RANKL) and osteoprotegerin (OPG). ^*^p < 0.05; ^***^p < 0.001, n = 5–6 per group and treatment.

To investigate whether osteoblast-specific overexpression of C5aR1 influenced osteoblast activity and bone remodeling, we performed *in vitro* and *in vivo* analyses. Western blot analysis confirmed considerably increased C5aR1 expression in primary osteoblasts derived from Col1a1-C5aR1 mice after inducing osteogenic differentiation (Fig 1A). Osteoblasts from WT and Col1a1-C5aR1 mice did not display any difference in proliferation (Fig 1B) or differentiation capacity as demonstrated by alkaline phosphatase staining (Fig 1C) and the expression of osteogenic differentiation markers (Fig 1D), also in the presence of C5a. Stimulation of WT and Col1a1-C5aR1 osteoblasts with C5a increased phosphorylation of extracellular signal-regulated kinase 1/2 (ERK1/2) and p38 mitogen-activated protein kinase (MAPK) (Fig 1E). In Col1a1-C5aR1 osteoblasts, phosphorylation of p38 was increased compared to WT osteoblasts, indicating that the receptor was biologically active. This was confirmed by significantly increased IL-6 and RANKL expression after stimulation of Col1a1-C5aR1 cells with C5a (Fig 1F).

Cross inspection and X-ray examination of the skeleton did not reveal any difference between 12-week-old and 55-week-old WT and Col1a1-C5aR1 mice (data not shown). μCT and histomorphometric analyses of the femurs demonstrated that C5aR1 overexpression on osteoblasts did not influence the bone mass, structural or cellular bone parameters or the bone formation rate neither in the cortical nor trabecular compartments, indicating that bone development and remodeling were not significantly affected (Table 2).

**Table 2:** Bone phenotype of WT and Col1a1-C5aR1 mice aged 12 and 55 weeks.

	Parameter	WT 12 weeks	Col1a1-C5aR1 12 weeks	WT 55 weeks	Col1a1-C5aR1 55 weeks
**cortex**	**TMD (g/mm**^**3**^**)**	1282 (±14)	1239 (±7)	1446 (±14)	1409 (±48)
	**C.Th (mm)**	0.20 (±0.01)	0.24 (±0.02)	0.21 (±0.02)	0.21 (±0.03)
**trabecular bone**	**BMD (g/mm**^**3**^**)**	200 (±27)	257 (±49)	193 (±12)	179 (±46)
	**Tb.N (1/mm)**	2.8 (±0.5)	3.4 (±0.8)	1.7 (±0.3)	1.8 (±0.3)
	**Tb.Th (mm)**	0.06 (±0.00)	0.06 (±0.01)	0.06 (±0.01)	0.06 (±0.01)
	**Tb.Sp (mm)**	0.19 (±0.02)	0.18 (±0.03)	0.24 (±0.02)	0.25 (±0.01)
	**Nb.Ob/B.Pm (1/mm)**	18.9 (±3.2)	21.5 (±2.6)	15.6 (±6.2)	11.0 (±3.3)^#^
	**Nb.Oc/B.Pm (1/mm)**	3.7 (±1.7)	3.1 (±1.9)	2.3 (±0.6)	2.3 (±0.7)
	**BFR/BS (μm**^**3**^**/μm**^**2**^**/t)**	0.5 (±0.04)	0.7 (±0.16)	0.2 (±0.22)	0.1 (±0.12)^#^

WT: wildtype, TMD: tissue mineral density, C.Th: cortical thickness, BMD: bone mineral density, Tb.N: trabecular number, Tb.Th: trabecular thickness, Tb.Sp: trabecular separation, Nb.Ob/B.Pm: number of osteoblasts per bone perimeter, Nb.Oc/B.Pm: number of osteoclasts per bone perimeter, BFR/BS: bone formation rate per bone surface. Data are shown as mean ± standard deviation. No data were found to be significantly different (p < 0.05) between strains.

^#^ p < 0.05 *vs*. 12-week-old Col1a1-C5aR1 mice, n = 5–8 per group.

### Osteoblast-specific overexpression of C5aR1 did not affect inflammation in response to fracture

**Fig 2:**
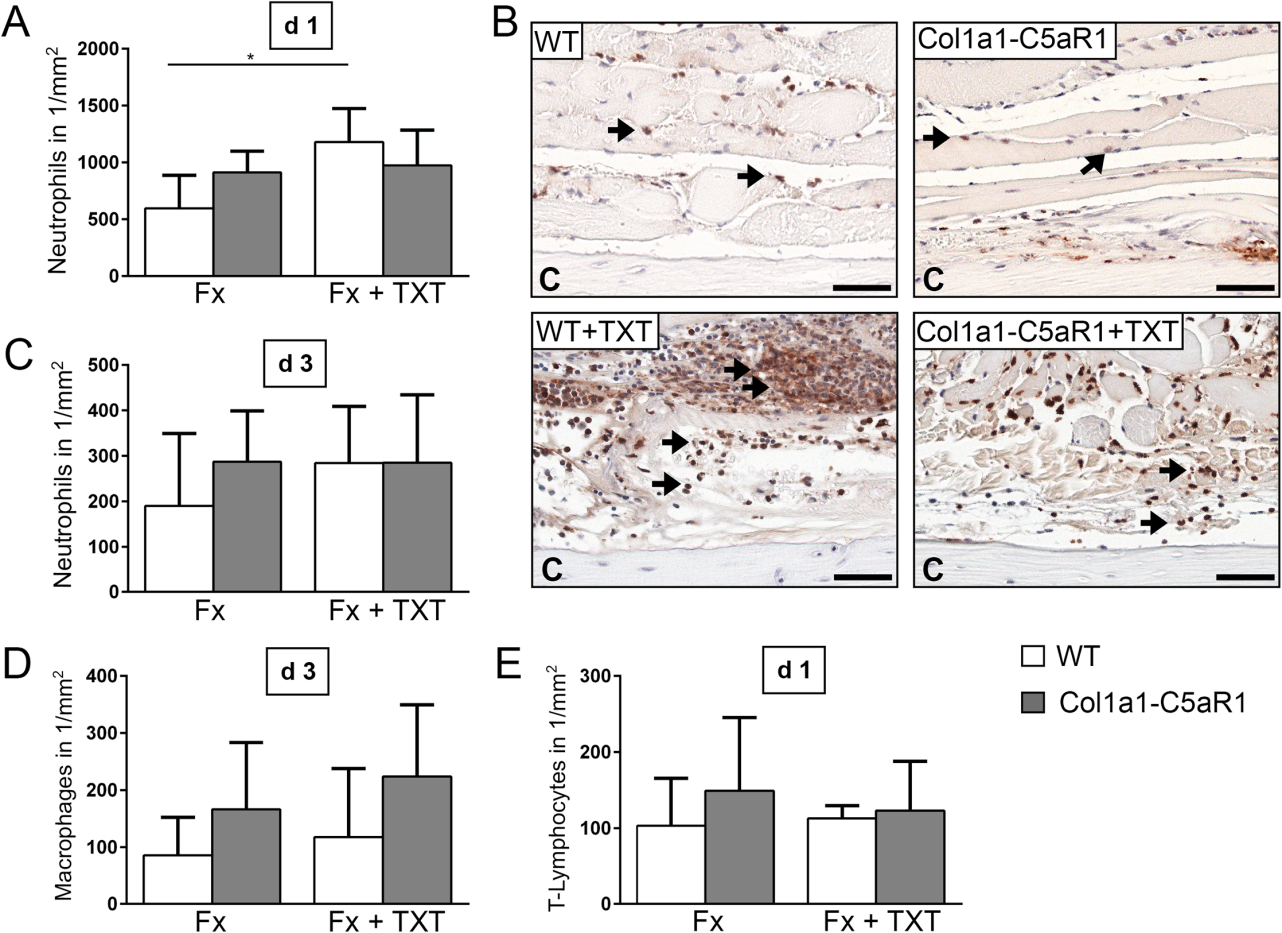
Number of inflammatory cells in the fracture callus of WT and Col1a1-C5aR mice. (A) Quantification of neutrophils (Ly-6G^+^) at 1 d, (B) representative immunohistological images of neutrophils 1 d after surgery and (C) quantification of neutrophils (Ly-6G^+^) 3 d after surgery. (D) Quantification of macrophages (F4/80^+^) 3 d and E) T-lymphocytes (CD8^+^) 1 d after surgery of wildtype (WT) and Col1a1-C5aR1 mice. Fx: mice with isolated fracture, Fx+TXT: mice with combined fracture and thoracic trauma (TXT). Arrows indicate some of the positively Ly-6G^+^ stained cells, C: cortex, scale bar 100 μm. ^*^p < 0.05, n = 6 per group.

To investigate whether osteoblasts modulate the early inflammation via C5aR1 at the fracture site, we analyzed the key inflammatory mediator IL-6 and the presence of immune cells locally in the fracture hematoma. C5a was locally and systemically generated after fracture in WT and Col1a1-C5aR1 mice without significant differences between the mouse strains (Table 3). In the isolated fracture model, IL-6 concentration (Table 3) and the numbers of neutrophils (Ly6G^+^ cells), macrophages (F4/80^+^ cells) and T-lymphocytes (CD8^+^ cells) (Fig 2) were not significantly altered in Col1a1-C5aR1 mice compared to WT mice.

**Table 3:** C5a and IL-6 concentrations in blood, BAL fluid and fracture hematoma 3 h after surgery.

		untreated mice	Fracture		fracture + TXT	
			WT	Col1a1-C5aR1	WT	Col1a1-C5aR1
**blood**	**C5a (ng/ml)**	0.8 (±0.6)	5.7 (±1.0)	5.3 (±1.5)	6.5 (±1.1)	6.3 (±1.6)
	**IL-6 (pg/ml)**	8.9 (±3.0)	42.8 (±20.3)	57.1 (±28.6)	154.2 (±59.0)*	188.2 (±38.4)*
**BAL fluid**	**C5a (ng/ml)**	8.6 (±1.2)	9.0 (±3.0)	8.6 (±1.4)	13.9 (±2.1)*	13.6 (±2.0)*
	**IL-6 (pg/ml)**	3.5 (±0.6)	3.2 (±3.9)	3.1 (±2.8)	123.7 (±55.6)*	218.7 (±161.0)*
**fracture hematoma**	**C5a (ng/mg protein)**	-	1.4 (±0.6)	1.2 (±0.6)	1.6 (±0.7)	2.0 (±1.0)
	**IL-6 (pg/mg protein)**	-	81.9 (±49.2)	80.4 (±51.8)	53.2 (±27.0)	35.3 (±26.4)

BAL: bronchoalveolar lavage, WT, wildtype, TXT: thorax trauma. Data are shown as mean ± standard deviation. C5a was analyzed in plasma and IL-6 was analyzed in serum.

^*^p < 0.05 *vs*. group without TXT, n = 5–6 per group.

Because C5a has been reported to trigger posttraumatic systemic inflammation after severe injury [23], we combined the fracture with an additional thoracic trauma. The combined trauma significantly enhanced serum IL-6 concentrations in WT and Col1a1-C5aR1 mice, indicating systemic inflammation (Table 3). In the BAL fluid, C5a and IL-6 were significantly enhanced in both genotypes (Table 3). The additional thoracic trauma did not influence the concentration of inflammatory mediators in the fracture hematoma (Table 3) but, according to our previous studies [14,15,19,24], increased the recruitment of neutrophils to the fracture site in WT mice (Fig 2). However, the osteoblast-specific overexpression of C5aR1 did not affect inflammatory parameters in the combined trauma model (Table 3, Fig 2).

### Osteoblast-specific overexpression of C5aR1 significantly impaired bone formation in the fracture callus

**Fig 3:**
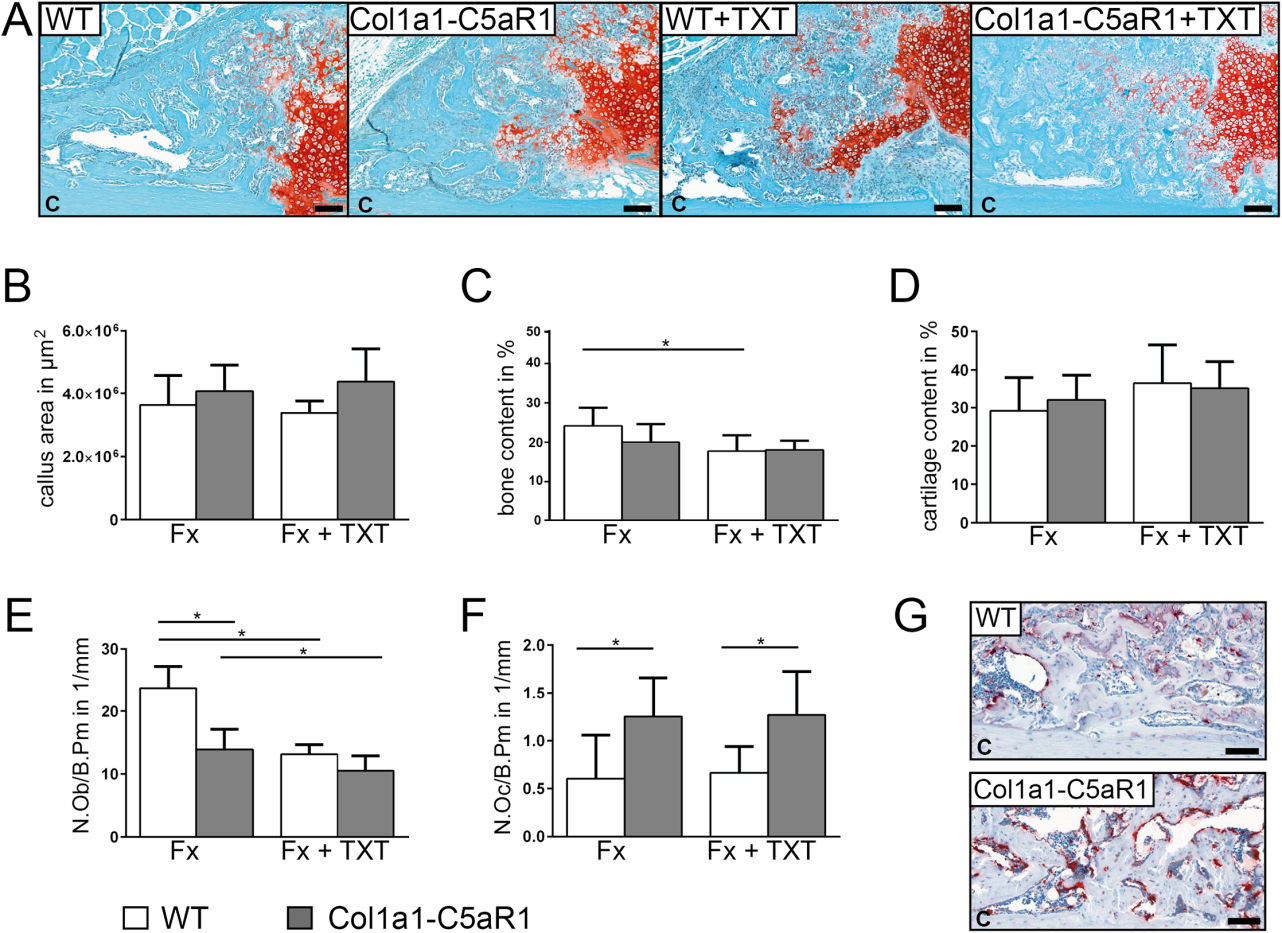
Fracture healing in WT and Col1a1-C5aR1 mice 14 d after surgery. (A) Representative histological images of wildtype (WT) and Col1a1-C5aR1 mice with isolated fracture and fracture with additional thoracic trauma (TXT). (B) Callus area, (C) amount of osseous tissue and (D) amount of cartilage. (E) Number of osteoblasts per bone perimeter (N.Ob/B.Pm) and (F) number of osteoclasts per bone perimeter (N.Oc/B.Pm) of WT and Col1a1-C5aR1 mice. (G) Representative tartrate-resistant acid phosphatase (TRAP) staining of fractured calli of WT and Col1a1-C5aR1 mice with isolated fracture. Fx: mice with isolated fracture, Fx+TXT: mice with combined fracture and thoracic trauma. C: cortex, scale bar 100 μm, ^*^p < 0.05, n = 6 per group.

**Fig 4:**
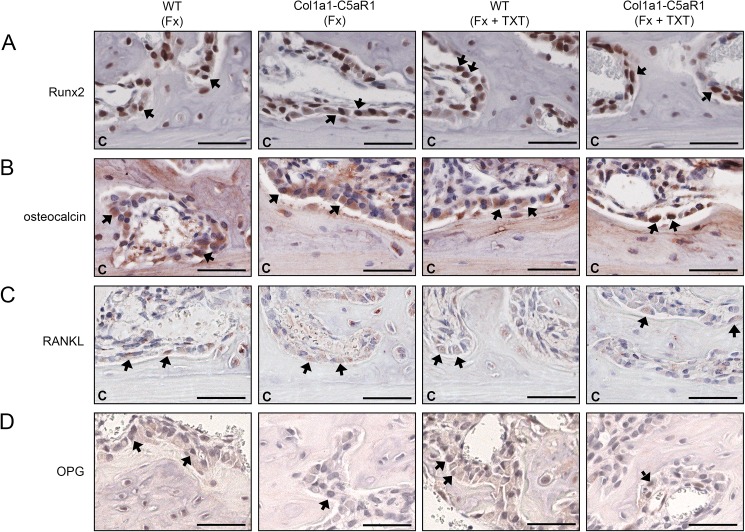
Representative immunohistological images of bone markers in the fracture callus of WT and Col1a1-C5aR1 mice 14 d after surgery. (A) Runx2, (B) osteocalcin, (C) receptor activator of nuclear factor-κB ligand (RANKL) and (D) osteoprotegerin (OPG). WT: wildtype, Fx: mice with isolated fracture, Fx+TXT: mice with combined fracture and thoracic trauma (TXT). Arrows indicate some of the positively stained cells, C: cortex, scale bar 100 μm.

To investigate callus formation, we analyzed the tissue composition in the repair phase on day 14, when callus tissue is abundantly formed by intramembranous and endochondral ossification. In the isolated fracture model, we did not find significant differences in the content of cartilage and bone between the genotypes (Fig 3A–3D). Notably, the number of osteoblasts was significantly decreased whereas the number of osteoclasts increased in the callus of mice with an osteoblast-specific C5aR1 overexpression (Fig 3E–3G). The additional thoracic trauma slightly reduced the amount of bone and decreased the number of osteoblasts in the callus without significant differences between both genotypes (Fig 3C and 3E). Immunostaining revealed intense staining for Runx2 and osteocalcin of osteoblasts on newly formed bone trabeculae in all groups without significant differences between genotypes, indicating undisturbed osteogenic differentiation (Fig 4). We did not detect significant differences in the expression of RANKL, but found less OPG-positive osteoblasts in mice with Col1a1-C5aR1 overexpression.

**Fig 5:**
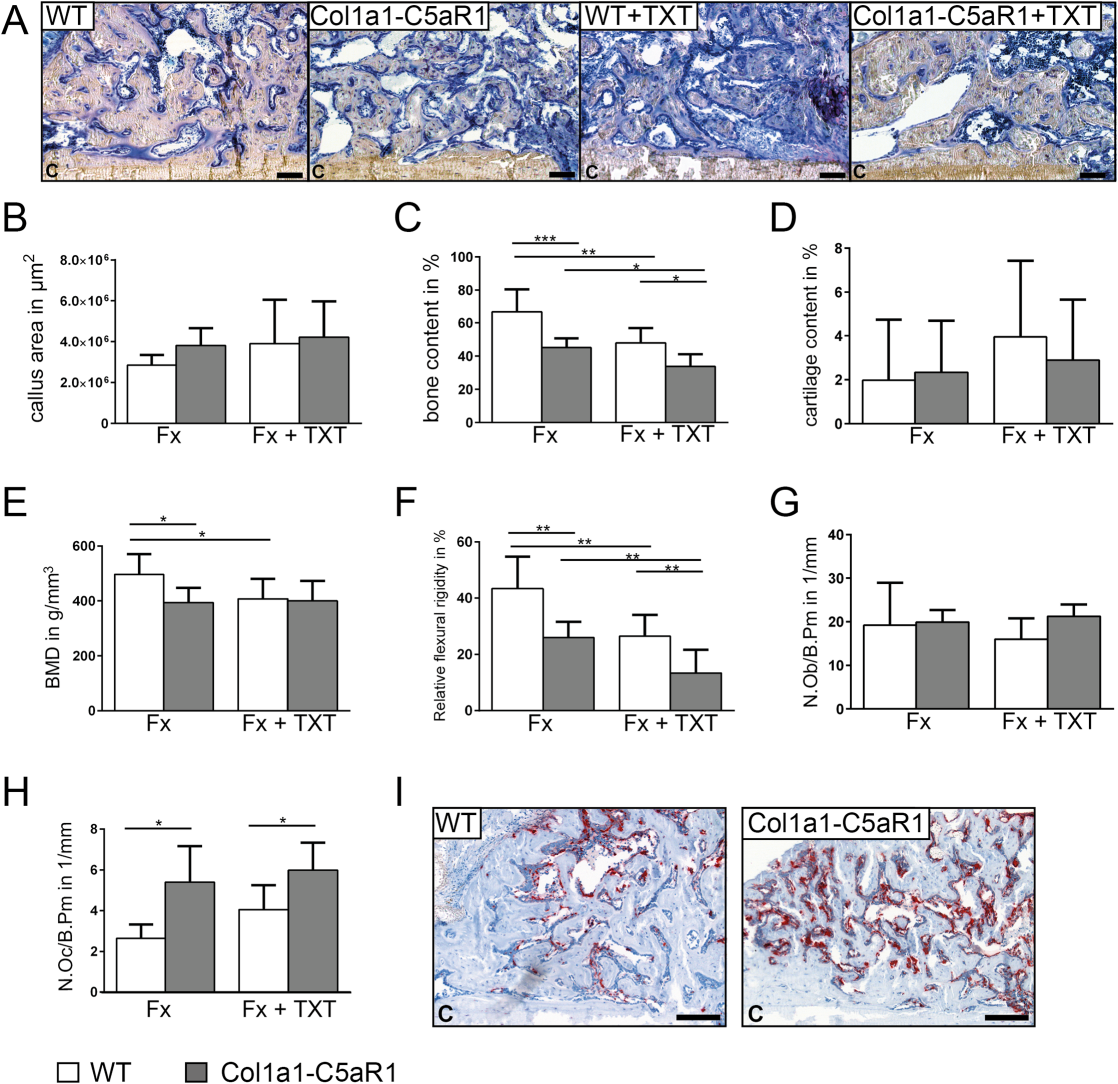
Fracture healing in WT and Col1a1-C5aR1 mice 21 d after surgery. (A) Representative histological images of wildtype (WT) and Col1a1-C5aR1 mice with isolated fracture and fracture with additional thoracic trauma (TXT). (B) Callus area, (C) amount of osseous tissue and (D) amount of cartilage. (E) Bone mineral density (BMD). (F) Relative flexural rigidity. (G) Number of osteoblasts per bone perimeter (N.Ob/B.Pm) and (H) number of osteoclasts per bone perimeter (N.Oc/B.Pm) of WT and Col1a1-C5aR mice. (I) Representative tartrate-resistant acid phosphatase (TRAP) staining of fractured calli of WT and Col1a1-C5aR1 mice with isolated fracture. Fx: mice with isolated fracture, Fx+TXT: mice with combined fracture and thoracic trauma. C: cortex, scale bar 100 μm, ^*^p < 0.05; ^**^p < 0.005; ^***^p < 0.001, n = 6–7 per group.

In WT mice with isolated fracture, most of the cartilage was transformed to bone after 21 days. However, in Col1a1-C5aR1 mice, bone content, BMD and the flexural rigidity were significantly reduced (Fig 5). We found more osteoclasts in the callus of Col1a1-C5aR1 mice, whereas osteoblast numbers were not different compared to WT mice (Fig 5G–5I). In Col1a1-C5aR1 mice, the additional thoracic trauma induced significantly more pronounced effects compared to WT mice as demonstrated by significantly reduced bone content and flexural rigidity (Fig 5C and 5F).

**Fig 6:**
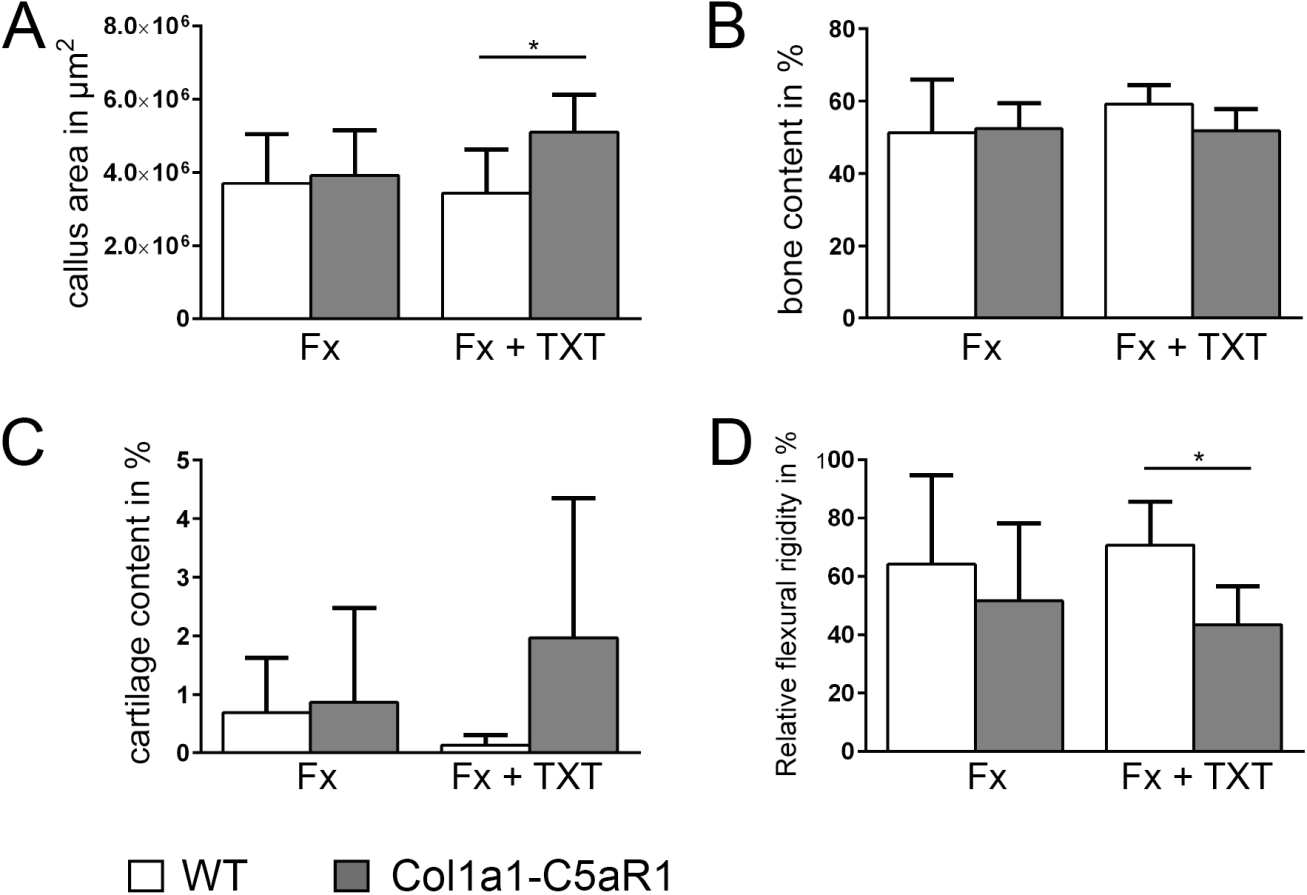
Fracture healing in WT and Col1a1-C5aR1 mice 25 d after surgery. (A) Callus area, (B) amount of osseous tissue, (C) amount of cartilage and (D) relative flexural rigidity of wildtype (WT) and Col1a1-C5aR1 mice. Fx: mice with isolated fracture, Fx+TXT: mice with combined fracture and thoracic trauma (TXT). ^*^p < 0.05, n = 6 per group.

After 25 days, fractured bones were completely healed in both genotypes with isolated fracture (Fig 6). However, at this time point, the strong effect of the thoracic trauma in Col1a1-C5aR1 mice was still clear as demonstrated by significantly reduced flexural rigidity of the fractured femurs, increased callus area and a tendential increase in the cartilaginous tissue area (Fig 6).

## Discussion

The objective of the present study was to unravel the role of the C5aR1 in osteoblasts regarding inflammation and bone formation post fracture using mice with an osteoblast-specific overexpression of C5aR1. Transgenic Col1a1-C5aR1 mice displayed normal bone development and turnover. However, fracture healing both in a model of uncomplicated fracture and in a model after severe additional trauma was considerably impaired in transgenic mice, indicating that C5aR1 signaling in osteoblasts may be crucially involved in bone formation under pathological conditions.

We first characterized the bone phenotype of Col1a1-C5aR1 mice to investigate whether C5aR1 overexpression affects bone development and turnover. The morphology of the skeleton and the density and structural parameters of cortical and trabecular bone were unaffected in both young and elderly Col1a1-C5aR1 mice. Col1a1-C5aR1 osteoblasts derived from these mice exhibited undisturbed proliferation and differentiation capacities, including after stimulation of the receptor with its ligand C5a. These results demonstrated that osteoblast-specific C5aR1 overexpression did not alter bone development or turnover. However, this did not exclude an important role for the C5aR1 under physiological conditions. It was demonstrated that osteoblasts produce the key complement protein C5 also in the absence of inflammatory stimuli and that osteoclasts can effectively cleave C5 to biologically active C5a [12]. Confirming this, Andrades et al. found C5 together with other complement components expressed in the bone growth plate and suggested that complement might contribute to the transformation of cartilage to bone during endochondral ossification [25]. Indeed, previous examination of the bone phenotype of C5-deficient mice demonstrated that the absence of C5 had an impact on cortical bone [2]. Twelve-week-old C5-deficient mice exhibited decreased bending stiffness and increased growth plates, confirming that C5a signaling could play a regulatory role in bone development, however, further studies are necessary to dissect the molecular mechanisms [2].

Confirming previous data that C5aR1 signaling induces a pro-inflammatory and osteoclastogenic response in osteoblasts [10,12], our results revealed that IL-6 and RANKL expression were significantly increased in Col1a1-C5aR1 osteoblasts in response to C5a. In agreement with findings in immune cells [26–28] and MSCs [11], we showed that the C5aR1 downstream signaling in osteoblasts involved ERK1/2 and p38 phosphorylation, which is known to regulate the expression of inflammatory cytokines, including IL-6 [27,29]. These data suggest that the C5aR1 axis in osteoblasts may be particularly important under inflammatory conditions. In accordance with this, it was shown that C5aR1-knockout mice were protected against osteoarthritis [30] and that bone loss in bacteria-induced periodontitis was associated with an increased C5aR1 activity in osteoblasts [31]. We previously demonstrated that osteoblasts strongly upregulated C5aR1 during the early phase after bone fracture [1]. Therefore, we hypothesized, that C5a, which is known to be abundantly generated locally and systemically in response to tissue injury [32–34], may induce an immune-modulatory response in osteoblasts during fracture healing. Our results revealed that the concentration of IL-6, a key cytokine in the local immune response after bone fracture and in posttraumatic systemic inflammation [35–38], was neither locally nor systemically altered in Col1a1-C5aR1 mice. Additionally, the recruitment of innate and adaptive immune cells to the fracture site was not significantly influenced, suggesting that C5aR1 signaling in osteoblasts did not modulate the early immune response after fracture. A possible reason may be the low number of osteoblasts compared to the high number of active immune cells during this early stage. However, bone healing was considerably disturbed in the later healing stages in Col1a1-C5aR1 mice. The bone content and mineral density and the mechanical properties of the fracture callus were significantly reduced. The reduced bone fraction may be primarily caused by a significantly increased number of osteoclasts observed after 14 and 21 days in the fracture callus of Col1a1-C5aR1 mice. These observations are in agreement with the present and previous *in vitro* data, demonstrating that C5a significantly enhanced the RANKL/OPG ratio in osteoblasts, thereby promoting osteoclastogenesis [12]. Immunohistochemistry did not reveal obvious differences in RANKL expression, but OPG expression was clearly reduced in the callus of Col1a1-C5aR1 mice. These results are confirmed by other authors demonstrating that C5aR1 is important for osteoclastogenesis [1,10,12]. Bone marrow cells from C5aR1-deficient mice and abrogating C5aR1 signaling in WT cells with a C5aR1 antibody resulted in decreased osteoclastogenesis, whereas stimulation of WT or C3-deficent bone marrow cells with C5a enhanced osteoclastogenesis [13]. Moreover, IL-6 is probably involved in the differentiation of osteoclasts regulated by complement, because IL-6 expression was decreased in bone marrow cells from C3aR/C5aR1-deficent mice [13]. In Col1a1-C5aR1 mice, osteoblast numbers in the fracture callus were reduced on day 14 but were unchanged on day 21 and the expression of the osteoblastic differentiation markers Runx2 and osteocalcin was unaltered, suggesting that osteoblast activity might not be considerably affected, which was confirmed by our *in vitro* data. However, this needs further investigation. Moreover, to the best of our knowledge, no literature data exist about the role of the second C5a receptor, C5aR2, in bone metabolism and fracture healing. Concluding, the data suggest that C5aR1 signaling in osteoblasts may crucially modulate osteoclast formation and activity and thereby increase bone resorption during fracture healing by inducing the expression of osteoclastic factors, including RANKL and IL-6.

We also investigated healing in a combined model of fracture and thoracic trauma, which induces a posttraumatic systemic inflammatory response, resulting in impaired fracture healing [14,15,19,24]. Previously, we demonstrated that blockade of the C5aR1 by a specific antagonist during the early inflammatory phase abolished the negative effects of the severe trauma on fracture healing, indicating that C5a abundantly generated during posttraumatic inflammation may crucially contribute to trauma-induced disturbed fracture healing [39]. The combined fracture and thoracic trauma induced a systemic posttraumatic inflammatory response and impaired bone healing in both WT and Col1a1-C5aR1 mice, as demonstrated by increased IL-6 serum levels and by significantly reduced bone content and mechanical properties of the fracture callus, respectively, in both mouse strains. The impairment of fracture healing by the additional thoracic trauma was more pronounced in mice with the osteoblast-specific overexpression of C5aR1, confirming the significance of the C5a/C5aR1 axis in osteoblasts during fracture healing. After 25 days, when the fractures were completely healed in WT mice, Col1a1-C5aR1 mice still exhibited a significantly reduced bending stiffness, suggesting a detrimental impact of C5aR1 signaling in trauma-induced impaired bone regeneration.

In conclusion, the present data together with our previous experiments [1,12,15,24,39,40] suggest a crucial role of the C5aR1 in fracture healing and implies that C5aR1 inhibition may be a suitable strategy to prevent fracture-healing complications particularly after severe trauma.
